# Understanding chronic pain in the ubiquitous community: the role of open data

**DOI:** 10.3389/fpain.2023.1208513

**Published:** 2023-08-11

**Authors:** Federico Monaco, Emmanouil Georgiadis, Kakia Chatsiou, Antonio Bonacaro

**Affiliations:** ^1^ Department of Medicine and Surgery, University of Parma, Parma, Italy; ^2^ School of Social Sciences and Humanities, University of Suffolk, Ipswich, United Kingdom; ^3^ School of Engineering, Arts, Science & Technology, University of Suffolk, Ipswich, United Kingdom; ^4^ School of Health and Sports Sciences, University of Suffolk, Ipswich, United Kingdom

**Keywords:** chronic pain, non-biomedical approach, open data, artificial intelligence, patient empowerment

## Abstract

The combined use of social media, open data, and Artificial Intelligence has the potential to support practitioners and empower patients/citizens living with persistent pain, both as local and online communities. Given the wide availability of digital technology today, both practitioners and interested individuals can be connected with virtual communities and can support each other from the comfort of their homes. Digital means may represent new avenues for exploring the complexity of the pain experience. Online interactions of patients, data on effective treatments, and data collected by wearable devices may represent an incredible source of psychological, sociological, and physiological pain-related information. Digital means might provide several solutions that enhance inclusiveness and motivate patients to share personal experiences, limiting the sense of isolation in both rural and metropolitan areas. Building on the consensus of the usefulness of social media in enhancing the understanding of persistent pain and related subjective experiences via online communities and networks, we provide relevant scenarios where the effectiveness and efficiency of healthcare delivery might be improved by the adoption of the digital technologies mentioned above and repeated subsequently. The aim of this perspective paper is to explore the potential of open data, social media, and Artificial Intelligence in improving the prevention and management of persistent pain by adopting innovative non-biomedical approaches.

## 1. Introduction

Chronic or persistent pain lasts for more than 3 months and it is estimated to affect 20% of the world’s population and account for up to 20% of physician visits (1, 2). Chronic pain is not only a significant symptom but also the root cause of the daily practices and discourses of sufferers that are centred around this critical condition (3).

Individuals with persistent pain may well be users of modern technologies such as social media, search engines, and wearable technologies to name a few. These types of technologies can potentially provide a wealth of information to healthcare databases and contribute to the development of innovative chronic pain prevention and management strategies. Moreover, electronic footprints provide important insights on lifestyle, allowing a better understanding of underlining chronic pain issues.

We postulate that persistent pain and related lifestyle repercussions are intertwined with digital everyday habits, as illustrated by five scenarios presented in this study. Such scenarios are provided as a supplement to this perspective paper, with the aim of clarifying the importance of interaction and inclusion for people living with persistent pain, at various social levels, through the use of technology and social networking.

Even though data anonymity is a requirement to be met during open data collection and analysis, recent methods of processing COVID-19 anonymised open data have demonstrated the effectiveness of this approach in designing innovative strategies to promote health. Therefore, we suggest that open data analysis of digital habits pertaining to persistent pain may become an equally effective strategy in the prevention and treatment of chronic pain.

## 2. Overview of non-biomedical approaches for chronic pain

Despite biomedical treatment being the predominant approach against chronic pain, there has been considerable debate with regard to its therapeutic appropriateness. Looking at the overcomplicated reality of individuals living with persistent pain (4), researchers have considered the need to include an array of therapeutic options to implement/personalise chronic pain treatment in order to provide an alternative framework to the mind–body dualism and to promote the adoption of holistic care (5).

The literature proposes cultural (6), social (7), and psychological solutions to relieve pain (8) and to overcome iatrogenic complications caused by medication (9). Integrating alternative therapeutic options into biomedical treatment would ignite new perspectives on conceptualising and managing pain through an innovative holistic ecosystem. A recent initiative looking at diverse solutions towards the prevention and treatment of chronic pain has suggested the need to move away from the current urbanised painogenic environment (10). This study describes various living conditions exacerbating pain, such as the frequent exposure to a multitude of physical and psychosocial determinants amplifying the frequency, severity, and length of undergoing pain and associated body sensations (11).

To provide a simplistic explanation, the current ways of tackling persistent pain through the biomedical approach seem to be too limited to appropriately handle such a multifaceted and complicated phenomenon. As Johnson and Woodall (12) state, “Living in modern society offers potential for health improvement through technological advances and digital advancements…”. Investing in advanced technology solutions aimed at preventing and managing chronic pain includes means such as cloud services, Artificial Intelligence, social networks, Internet of Things (IoT), and so on. Such means are explained below.

## 3. Open data for the advancement of chronic pain research

A better understanding of the needs of individuals affected by persistent pain implies the acquisition of their personal perspectives. Focusing on how people think and feel about pain, including on the opinions of those surrounding them, such as caregivers, friends, neighbours, and so on, may offer us new perspectives on the impact of the painful experience, including personal meaning and related daily practices. This seems to provide important opportunities for appreciating the complexity of chronic pain from a holistic perspective.

Adequate and comprehensive data such as Big Data may contribute to improving the quality of previously acquired minimal datasets (13). Understanding which ontological approach and related data elements (14) would be more suitable for data sharing could facilitate the study of behavioural patterns appearing in social networks and the creation of digital citizen labs (15) where open discussions may foster public engagement on an important topic. A valuable example may be given by the introduction of effective ways to support individuals affected by persistent pain. Promoting the adoption of positive thinking techniques and allowing practitioners to share successful stories on social networks might support the formulation of self-management strategies (16).

Recent developments on Open Data management in delivering responses to the COVID-19 pandemic have demonstrated how helpful this information is in improving evidence-based practice. Automated data collection, databasing, and data processing through the adoption of forms of Artificial Intelligence and machine learning (17) may be critical for effectively enhancing disease monitoring and for delivering high-quality care in a timely manner (18).

## 4. Open data helping ubiquitous communities of people living with chronic pain

Capturing the connections and types of interactions of online social life used by patients with chronic pain is not a new technique (19), especially when it comes to empowerment (20), given that online support groups tend to prefer interaction in a virtual environment (21). For a long time, the role of social interaction has been deemed important among individuals with persistent pain, showing the potential to meet the information needs and expectations of users (22). Health-related issues have been discussed in open and innovative platforms (23) as part of European Union–funded projects (https://cordis.europa.eu/project/id/688670). For example, the links between menopause and chronic pain (24) were identified through the platform named GENNEV, subsequently allowing the delivery of telehealth and coaching services (https://www.gennev.com/). Despite its success, GENNEV lacks an essential feature, which is the opportunity for peer interaction.

In other studies, such online interaction is considered an important component of tackling chronic pain (25). It seems that patients with chronic pain may be able to overcome the stigma and invisibility of persistent pain through mutual online empowerment (26). For example, the possibility of being visible and of having unlimited opportunities for conversation with a wider audience may have important positive effects (27, 28).

## 5. Open data and community resilience

Despite the obvious concerns about data privacy, the case of open data sharing during the COVID-19 pandemic shed light on how data may suggest the adoption of new collective and individual tasks/habits. Online networks and communities provide a digital infrastructure, where each member may share useful data through peer-to-peer interactions, and favour positive impacts on chronic pain (29).

Such data types are of great value for communities and researchers, especially when they allow them to explore daily routines around pain and related habits and to gain a better understanding of community practices that are put in place for the benefit of both patients and stakeholders. Such communities seem to grow by sharing common needs, values, and interests. Furthermore, a variety of relevant healthcare professionals may be invited to be a part of such communities, ensuring further benefits to their members.

## 6. Ubiquitous communities: when patients, caregivers, and experts come together

Various examples may prove the potential of public engagement in tailoring innovative evidence-based practice. In the mid-1980s, AIDS prevention campaigns were driven by the successful integration of public awareness into biomedical research (30). Similarly, not-for-profit organisations such as the Cochrane Collaboration or James Lind Alliance offer examples of possible collaborations among patients and healthcare providers or researchers.

Making use of the vast potential offered by the World Wide Web (WWW), today’s technology users provide an unprecedented ubiquity of resources and digital infrastructures that may connect people who share common interests and goals around the world. Enhanced opportunities that serve to connect people may be utilised similarly in advancing health collaborative practices. Open data sharing is key in this regard, especially when information is drawn from different sources and combined to get a more comprehensive picture of the experiences of users suffering from persistent pain (31).

The EU-funded project Opencare (http://opencare.cc/) is part of the Collective Awareness Platforms for Sustainability and Social Innovation (CAPSSI), which provides a valuable example of such enhanced interconnectivity. This initiative unveils the potential of allowing people affected by chronic pain in Europe to engage in discussions about the kind of support they need and to undertake initiatives to reach out to local governments and health authorities, thus eliciting a higher quality of care.

Digital communities, along with the support of experts, may potentially play a more active role in supporting individuals suffering from persistent pain (32). Health-promoting infrastructures (HPIs), such as networks aimed at finding the availability of health expertise and at promoting solutions for global health problems, are a relevant example of such digital communities (33).

We use “ubiquitous computing” as an umbrella term that describes a plethora of technologies able to support research and healthcare delivery while providing a formidable health data monitoring and surveillance opportunity for shaping everyday living healthcare provision plans (34). This goal becomes even more relevant when addressing health issues, especially in complex urban contexts (35). We acknowledge the need for the adoption of such a concept that includes important design features (36) based on the increasingly common use of wearable devices (37).

**Figure 1:**
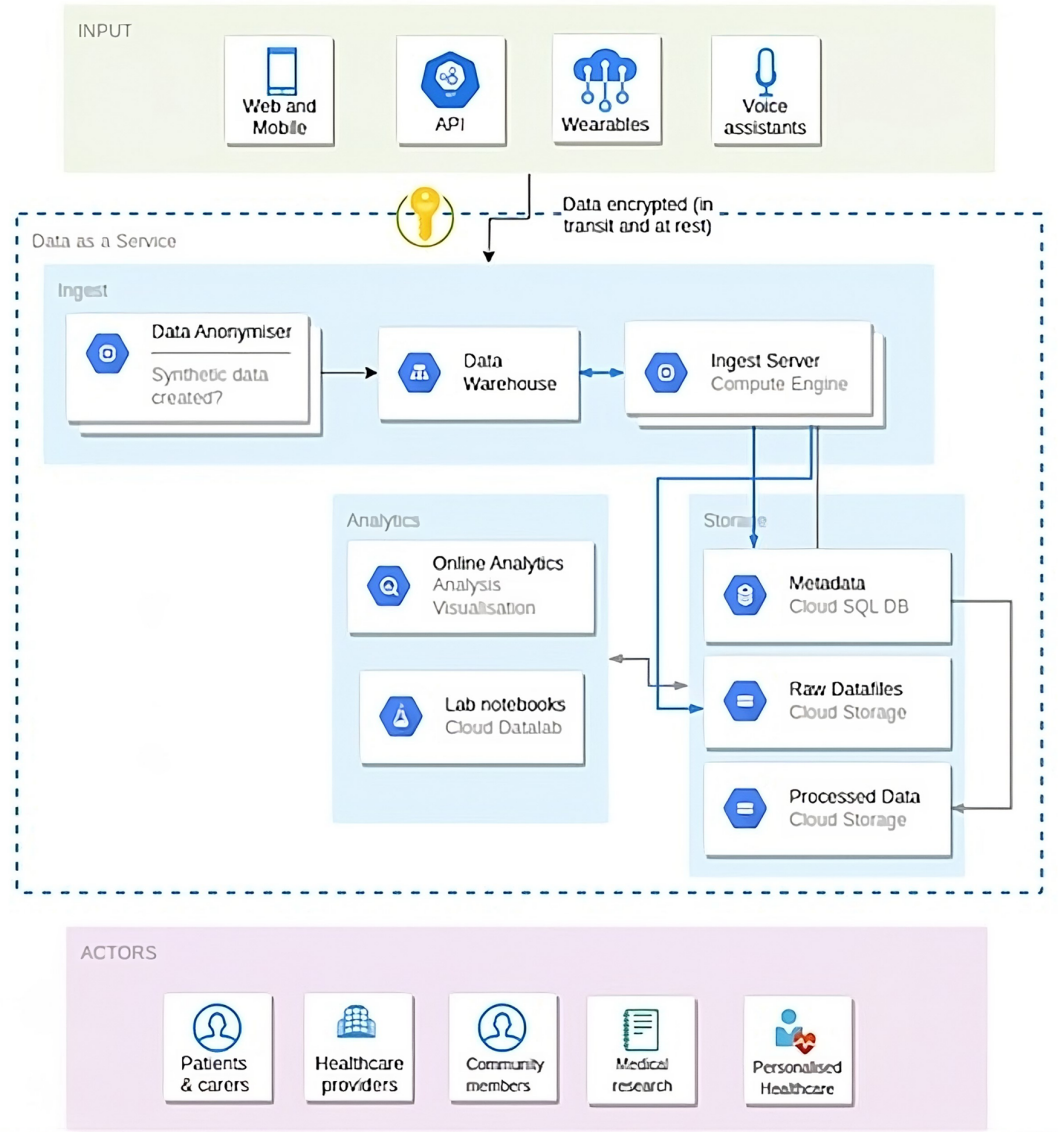
Blueprint of information architecture and a data sharing model for open patient data 161 ecosystems.

Equally, social network sites (SNSs), such as social media create a platform for the exchange of accurate information among peers belonging to virtual communities (38). Nonetheless, using social media to address chronic pain issues and provide relevant related services (40) implies fulfilling ethical and professional requirements (39). Further research and subsequent regulations on these aspects are deemed crucial in order to proceed without encountering any stumbling blocks or drawbacks. An example of a suggested model for this data analysis is presented in Figure 1.

## 7. A foresight approach with multiple scenarios

To build our case, five descriptive scenarios were developed concerning people affected by chronic pain. These scenarios include examples of patients producing data by blogging, using social networking, and adopting wearable technologies and sensors. The heterogeneity of the conditions described in these scenarios offers a constellation of narratives that will be useful in predicting future opportunities in terms of interoperability among patients, healthcare providers, digital experts, and policymakers. Scripts suggest ways to acquire data and the advantages of adopting an open data approach where patients, researchers, and practitioners may access and exchange such data.

Ubiquitous communities may gain a deeper understanding of current evidence-based strategies to tackle persistent pain through the use of relevant digital infrastructure while receiving guidance from subject experts, as highlighted in the five attached scenarios (Appendix 1). This would fuel local and global statistics on chronic pain and lead to the provision of more effective healthcare services. Similarly, policymakers might access such data and allocate proportionate human and financial resources accordingly.

Each scenario presents a different illness from a holistic perspective, and they were developed jointly by all authors. In addition to physical, psychosocial, and spiritual elements, the role of digital technologies is emphasised. Each scenario is complemented with an AI-generated picture of the interested patient, thus amplifying the impact of the narration. The five scenarios are based on the following illnesses: Parkinson’s disease, diabetes, knee pain, post-traumatic stress disorder, and neuropathic scar pain following a caesarean section.

## 8. Conclusions

The successful management of the COVID-19 pandemic based on an open data approach and community engagement paved the way for new avenues in tackling chronic pain and other equally important silent pandemics.

In this perspective paper, we propose an innovative approach for the prevention and management of chronic pain through the adoption of community-driven solutions based on open data. Chronic pain is a complicated and idiosyncratic phenomenon, which is very often de-contextualised from the everyday living experiences of patients and caregivers. Chronic pain is an area where a standardised biomedical approach based on drug administration is very often predominant. Such pain is frequently associated with a lack of control, uncertainty, ineffective treatment, high cost, and a lower quality of life. Open data generated by users, caregivers, healthcare professionals, and digital infrastructure might provide insights on how to effectively reshape healthcare practices on the basis of the daily habits and the real needs and expectations of patients and caregivers and also provide real-time critical mass data for performing more accurate research in the field.

This information might also be used as a form of online support and to provide instant feedback, on the effectiveness of screening and rehabilitation programs, patients’ medication compliance, average treatment duration, and behavioural trends.

Including users as active producers of data would help provide community-enabled solutions in tackling chronic pain, which would, in turn, empower them as co-creators of healthcare plans and services.

Today, modern technology makes possible the exploration of innovative solutions based on home monitoring and open data analysis.

In the five attached real-life scenarios, we depict situations where digital infrastructures may be used in cases of patients with chronic pain for sharing clinical information in an accurate and transparent way (41).

Further research is recommended in this area, which should aim at creating bottom-up solutions (i.e., ubiquitous communities—healthcare authorities) that would ideally include the issues of interoperability, data privacy, and digital divide (42, 43).

## Data Availability

The original contributions presented in the study are included in the article, further inquiries can be directed to the corresponding author.

## Appendix 1

### Scenarios

#### Scenario 1: Parkinson’s disease

Graham is 73; he was diagnosed with Parkinson’s disease 2 years ago. John has also a history with silicosis because of his long-term job as a miner, which caused permanent inflammation and fibrosis. Graham can hardly walk now because of an accident that happened many years ago in a mine and needs a walking frame to move from one room to the other.

Graham lives with his son Adam, who is divorced with two daughters aged 10 and 15. Graham’s condition has made them very active caregivers. Adam is an IT professional and he has set up an IoT (i.e., Alexa) system at home in order to stay in contact with his father and check what he does during the day when he is alone at home.

Therefore, Graham can be tracked, thanks to a Radio Frequency Identification (RFID) wristwatch allowing Adam to discuss with the personnel at the hospital Graham’s mobility and whether his father is performing the daily physical cardio and chest exercises prescribed to improve his psychological condition and wellbeing. RFID has proved to be a very important means of clarifying the levels of Graham’s sedentariness and helping to reduce the risk of depression. His family has already explored other means to increase his mobility, such as having a personal trainer or a nurse at home, but Graham likes to spend time alone with his hobbies and gets nervous when obliged to attend an exercise plan under the supervision of others. He likes to spend his time with his granddaughters, but this is not always possible because of their schooling and social endeavours.

#### Scenario 2: diabetes

Susan is 56 and suffers from diabetes. Her situation is getting more complicated now because of a foot ulcer that is causing chronic pain. She lives on her own as her husband left her 3 years ago when he fell out with the general practitioner supporting Susan. Apparently, he believed in holistic methods of treatment, and he felt that he could not handle the traditional biomedical approaches that Susan chose to follow for her condition.

Susan is a blogger publishing about type 2 diabetes and does a great job in providing information to, and networking for, the patient community about this disease condition. She keeps herself updated with evidence-based research and likes to disseminate knowledge only after she cross-references scientific studies during her meetings with the physician and the manager of the program that she follows. She posts on her blog every week and many people interact with her online.

Online discussions have supported other patients and helped improve their type 2 diabetes condition. Susan has been repeatedly asked for advice by other patients on ways to handle pain, but she feels reluctant to share suggestions on this topic as she is not an expert. Once every year, she organises a meeting for patients in her area to which physicians and nurses are invited. This helps her to gain a lot of attention on social media, and this event is growing in popularity year after year. Thanks to a funded therapeutic protocol, she is participating in a program for monitoring her diabetes, which runs by using experimental sensors for continuous glucose monitoring. Susan has made arrangements for the results of that therapeutic protocol to be disseminated through her blog as well.

#### Scenario 3: knee pain

Linda is 41 and has been visually impaired since birth. Unfortunately, she suffers from chronic knee pain and several injuries because of a history of falls and accidents. Linda lives with her family, which comprises her husband and two daughters who are 13 and 8 years of age. She loves to go to the Opera, and to satiate this desire, a charity association has agreed to take her by car to the local theatre, while her husband takes care of their daughters.

Linda’s health is also challenged by obesity because of an uncontrolled appetite for food, which leads to stomach pain after recurrent binge episodes when she is alone at home listening to audiobooks and operas and eating without control. Linda is enrolled in a special program that involves the following different topics:

(a) She is learning to instruct a tablet equipped with a software specifically designed to help her coping with her visual impairment.
(b) She meets with a self-help group weekly online, discussing ways to deal with her eating disorder.
(c) She gets at-home support from a physiotherapist, who treats her knee arthritis with low-impact exercises to maintain the joints and strengthen her muscles.

Linda wears a sensor that is able to monitor her walking (counting the number of steps, sedentariness, etc.), and it transmits data to the rehabilitation centre, where statistical data about her fitness and physiotherapy programme are stored.

#### Scenario 4: mental health (PTSD)

Fedir is a 23-year-old Ukrainian suffering from post-traumatic stress disorder (PTSD). His mental health issues started after he was wounded in a conflict as a soldier in the battle of Donbas. He is now following a rehabilitation program, trying to get support for his brain injuries.

He wears a heart monitor as well as a neurofeedback device. However, sometimes Fedir believes that his rehabilitation program is not assisting him at all, and he frequently fears for the safety of his online data. Therein lies his problem. Because of these fears, he removes his sensors, thus ceasing the monitoring process.

Fedir is now following an additional program through weekly meetings with a mental health counsellor, who tries to convince him about the need to not remove his sensors. This is crucial, as data from sensors are transmitted to a centre that runs a research protocol on PTSD involving patients from countries around Europe, and this centre is harvesting data that aim to confirm the most beneficial treatment for this difficult mental condition.

#### Scenario 5: neuropathic scar pain following Caesarean section

For more than 3 months, Liz, a 39-year-old, has been suffering from neuropathic scar pain following Caesarean section. She lives with her family in the countryside, 45 km away from the nearest town. She has two children, while her husband is a salesman who spends most of his time travelling for work, returning home only on weekends. Unfortunately, Liz cannot count on any other relatives or friends to support her during her daily chores, and she often feels overwhelmed because of her pain. Whenever she experiences a pain flare, Liz gets anxious and she feels that she cannot provide proper care to her children.

Liz’s physician lives in the town, but she finds it difficult to talk to the members of the clinic because of the long waiting times whenever she calls the clinic for support. She has been advised to take medication (paracetamol) when her pain gets intense and to book a medical appointment over the phone. The next available slot for a medical consultation is in 3 months’ time, and Liz has been exploring a better strategy to manage her pain by looking for available online support, but with limited success. A doctor in the hospital heard about Liz and organised an online medical consultation with her, which went successfully, and this has been repeated once every month since then. He also suggested that Liz start online sessions with mental health experts in her local area to get support for her mental health as well.
